# The first birthday of OpenAI’s Sora: A promising but cautious future in medicine

**DOI:** 10.1097/JS9.0000000000002432

**Published:** 2025-05-12

**Authors:** Sike He, Yuan Wang, Ziqi Li, Nan Jiang, Guangxi Sun

**Affiliations:** a Department of Urology, Institute of Urology, West China Hospital, Sichuan University, Chengdu, China; b College of Management Science, Chengdu University of Technology, Chengdu, China

*Dear Editor*,

On 15 February 2024, Sora, a text-to-video generative artificial intelligence (AI) model developed by OpenAI was released (https://openai.com/sora/). Sora is able to generate realistic and imaginative visual scenes from text input, which represents a milestone in AI and further bridges the gap between language processing and visualization creation by using the combination of Large Language Models, deep learning, computer vision, and natural language processing^[^1^]^. The emergence of Sora further expands the input-output patterns in AI models (e.g., text, voice, image, and video)^[^2-4^]^. During the past year, Sora has already attracted a huge number of users, though it is only accessible for selected regions. This innovative AI tool shows exciting potential to revolutionize the entire field of medicine and healthcare, especially reshaping medical education and patient care in this era of precise and personalized medicine^[^4,5^]^. However, some technical, legal, security, and ethical concerns have also been raised to keep researchers cautious^[^1,6^]^.

In this brief report, we conducted a bibliometric study focusing on literature related to Sora published since it is born, aiming to explore the current trends and future implications in this topic.

A comprehensive online search was performed in PubMed using the keywords “Sora,” “text-to-video generative artificial intelligence,” and “AI-generated video.” Only studies published before 15 February 2025 are eligible for further investigation.

After screening, a total of 15 studies were included and published in 10 journals (Table 1). The number of publications was relatively low in total and per month (Fig. 1A). Regarding literature type, Letter occupies the vast majority, followed by Review, and there is only one article (Fig. 1B). These publications focus on various subjects, “surgery” (neurosurgery, plastic surgery, and urology) is the most popular, followed by “general medicine” (Fig. 1C). The keywords network conducted by VOSviewer (Version 1.6.20) shows some topics that are highlighted (e.g., “education,” “neurosurgery,” and “ethical concern”) (Fig. 1D).

**Figure 1.**
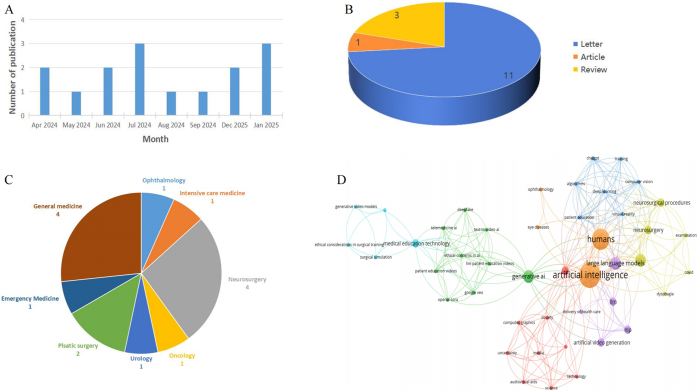
(A) Number of Sora publications per month. (B) Literature-type distribution is Sora publications. (C) Subjects focused by Sora publications. (D) Collaborative network of keywords.

**Table 1:** Summary of current literature about Sora in medicine

Study title	Journal	Publication time	Main opinion
Concerns with OpenAI’s Sora in Medicine	Annals of Biomedical Engineering	2024.4	(6)
OpenAI’s Sora in medicine: revolutionary advances in generative artificial intelligence for healthcare	Irish Journal of Medical Science	2024.4	(1)(2)(4)
OpenAI’s Sora in ophthalmology: revolutionary generative AI in eye health	Eye	2024.5	(1)(2)(3)
Text-to-video generative artificial intelligence: Sora in neurosurgery	Neurosurgical Review	2024.6	(2)(3)(4)
OpenAI’s Sora in intensive care medicine: opportunities and challenges	International Journal of Surgery	2024.6	(1)(2)(3)(5)
Text-to-video generative artificial intelligence: Sora in neurosurgery: correspondence	Neurosurgical Review	2024.7	(6)
OpenAI’s Sora: It’s time to update the dissemination methods of medical findings	International Journal of Surgery	2024.7	(1)(3)(4)(5)
AI-Driven neurosurgery with Sora: applications and complexities	International Journal of Surgery	2024.7	(1)(2)(3)(5)
From text to video: What will OpenAI’s Sora bring to the oncologic field?	International Journal of Surgery	2024.8	(2)(3)(5)
Application of transformer architectures in generative video modeling for neurosurgical education	International Journal of Computer Assisted Radiology and Surgery	2024.9	(1)
Expanding the horizons of OpenAI’s Sora: Unleashing potential in urology	Asian Journal of Surgery	2024.12	(1)(2)(3)
Text-to-Video Models and Sora in Plastic Surgery: Pearls, Pitfalls, and Prospectives	Aesthetic Plastic Surgery	2024.12	(1)(2)(3)(5)
Using OpenAI’s Text-to-video Model Sora to Generate Cardiopulmonary Resuscitation Content	Resuscitation	2025.1	(1)
OpenAI’s Sora and Google’s Veo 2 in Action: A Narrative Review of Artificial Intelligence-driven Video Generation Models Transforming Healthcare	Cureus	2025.1	(1)(2)(3)(6)
Commentary on: “Text-to-Video Models and Sora in Plastic Surgery: Pearls, Pitfalls, and Prospectives”	Aesthetic Plastic Surgery	2025.1	NA

(1) medical education; (2) patient education; (3) clinical practice; (4) public health; (5) academic communication; (6) ethical concern; NA: not applicable.

Compared with another famous AI tool, ChatGPT, published in November 2022, the number of publications in Sora is significantly lower without an upward trend month by month^[^7^]^. In fact, despite sharing the same developer (OpenAI), ChatGPT and Sora diverge substantially in their architectural frameworks and operational functionalities as generative models (Table 2). Totally speaking, ChatGPT excels in language-based interaction contexts, while Sora specializes in visual generation and motion presentation scenarios. A large number of letters indicated that researchers are currently doing preliminary explorations and outlooks in this field, for example, using Sora to generate cardiopulmonary resuscitation content^[^8^]^, or summarizing the potential application of Sora in eye health^[^9^]^. Notably, journals across various specialties showed great interest in the Sora applications in respective fields, including neurosurgery, urology, plastic surgery, emergency medicine, and so on. More original reports based on larger cohorts are needed. Intriguingly, issues related to “surgery” are commonly mentioned in these publications. The powerful simulation capability can help surgeons compare different surgical plans, and create surgical procedure videos for medical students to better understand and master anatomical structures and surgical techniques^[^10,11^]^. Then, treatment approaches can be simply and clearly explained to patients to aid them in preoperative preparation and postoperative recovery^[^10^]^. In short, Sora can provide tremendous support for the perioperative period.

**Table 2:** The comparison between ChatGPT and Sora

	ChatGPT	Sora
Developer	OpenAI	OpenAI
Primary function	Text generation, conversation, code, Q&A	Video generation (high-quality videos from text prompts)
Core technology	GPT-based large language model	Diffusion Model + Transformer
Input	Text	Text (and potentially images/video)
Output	Text	Video
Training data	Massive text data (books, web pages, conversations)	Video and image datasets
Applications	Customer service, writing assistance, programming, education	Film production, advertising, gaming, art
Interactivity	High (multi-turn dialogue)	Low (one-way generation, requires prompt tuning)

Here, we describe the six key focuses in Sora literature:

- Public health: Video is a useful tool to spread basic medical knowledge to the public due to its audio and visual properties. With the development of online platforms and social media, the integration of videos generated by Sora and online media has the potential to be effective in public health education, especially during pandemics, such as COVID-19 and Monkeypox^[^12^]^.
- Medical education: Sora shows considerable ability in medical training and education since it can provide interactive learning patterns, immersive simulations for skill training, and various clinical scenarios. By real-life cases presentation, surgical procedures, instrument handling, and nursing methods, medical staff obtain a more profound grasp of diagnostic and therapeutic approaches to diseases, thereby enhancing their skill and professional acumen^[^4,13^]^.
- Patient education: Sora can be used to help patients and their families better understand medical concepts. By explaining the disease features through video format, patients’ comprehension of their conditions is improved, thereby fostering adherence to treatment plans. Then, some self-care instructions, such as improved inhaler technique, or rehabilitation exercises, can also be taught by Sora^[^4^]^.
- Clinical practice: Sora can act as an assistant to help clinicians in decision-making, such as patients’ assessment and surgical plans. Additionally, Sora can significantly improve telemedicine and remote healthcare by creating medical videos that support teleconsultations, remote monitoring, and real-time guidance. This is especially valuable in underserved and developing areas^[^6,11^]^.
- Academic communication: Different from traditional publications and conferences, Sora can transform text to videos to vividly illustrate the research process, experimental results, and conclusions. In addition, Sora can spread the newest researches through online platforms to improve knowledge accessibility and audience reach, and finally, markedly enhance the efficiency and efficacy of academic communication^[^13^]^.
- Ethical concern: Some risks and concerns are faced by researchers and users of Sora. When AI-generated videos are utilized in healthcare settings, they may produce incorrect medical opinions and contribute to the wide spread of false information. Misleading caused by AI can induce invalid medical treatments, overstate the efficacy of medical interventions, and even violate legal limits. These risks can lead to mistakes in diagnosis and treatment, further harm patient’s interest, and eventually damage public confidence in medical workers and organizations^[^1,11^]^.

Here we present some cases using Sora to generate videos in different scenarios: Case 1: “wearing N95 mask”; Case 2: “demonstrating chest compressions”; Case 3: “demonstrating surgical suture” (the videos generated by Sora can be checked in Supplementary Materials, available at: http://links.lww.com/JS9/E124, http://links.lww.com/JS9/E125, http://links.lww.com/JS9/E126). Sora successfully generated three videos, but none of them accurately described the settings, which were similar to the study by Fijačko *et al*^[^8^]^. These cases highlighted shortcomings of the applicability of such AI-generated videos.

This study overviews the first year of Sora and elucidates both its wide use and limitations in different aspects of medicine. Sora presents a fabulous advancement in AI and it may reshape the future healthcare landscape. However, potentials, expectations, risks, and concerns will always coexist. As its applications are continuously expanding, more vigilance and optimization are vital.

## Data Availability

The data underlying this article will be shared by the corresponding author on reasonable request.
